# Supplementary material for: Genome-wide identification, evolution, expression, and alternative splicing profiles of peroxiredoxin genes in cotton
Source: PeerJ. 2021 Jan 18;9:e10685. doi: 10.7717/peerj.10685 (PMC7819121; doi:10.7717/peerj.10685)
Supplement: Figure S1 — Cotton selection G. hirsutum represents type PRXII, ”*” means that the residues in that column are identical in all sequences in the alignment, ”:” means that conserved substitutions have been observed, The sequences alignment was done with ClustalW tool, and the default setting is slow mode. [file peerj-09-10685-s001.pdf]

```
PRXIIF  AT3G06050       [59]SSKFSTTPLSDIFKGKKVVIFGLPGAYTGVCSQQHVPSYKSHIDKFKAKGIDSVICVSVNDPFAINGWAEKLGAKDAIE
PRXIIF  Os01g0266600    [56]ATNFSTTPLKDIFHGKKVVIFGLPGAYTGVCSQAHVPSYKNNIDKLKAKGVDSVICVSVNDPYALNGWAEKLQAKDAIE
PRXIIF  GhPRX6-A        [56]TSNFSTTSVNDIFKGKKVVIFGLPGAYTGVCSQQHVPSYKKNIDKFKAKRIDSVICVAINDPYVMNAWADKLQAKDVIE
PRXIIF  GhPRX14-D       [56]TSNFSTTSVNDIFKGKKVVIFGLPGAYTGVCSQQHVPSYKKNIDKFKAKGIDSVICVAVNDPYVMNAWADKLQAKDVIE
PRXIIE  AT3G52960       [91]TGDVKTVTVSSLTAGKKTILFAVPGAFTPTCSQKHVPGFVSKAGELRSKGIDVIACISVNDAFVMEAWRKDLGIND-EV
PRXIIE  Os06g0625500    [89]DGELKTVTVRDLTAGKKVVLFAVPGAFTPTCTQKHVPGFVAKAGELRAKGVDAVACVSVNDAFVMRAWKESLGVGD-EV
PRXIIE  Os02g0192700    [81]DGELKTVTVAELTAGRKAVLFAVPGAFTPTCSQKHLPGFIEKAGELHAKGVDAIACVSVNDAFVMRAWKESLGLGDADV
PRXIIE  GhPRX1-A        [84]DGELQTTTISSLTAGKKAVIFAVPGAFTPTCSQKHLPGFVEKSGELKAKGVNTIACVSVNDAFVMRAWKENLGIKD-EV
PRXIIE  GhPRX8-D        [84]DGELQTTTISSLTAGKKTVIFAVPGAFTPTCSQKHLPGFVEKSGELKAKGVNTIACVSVNDAFVMRAWKENLGIKD-EV
PRXIID  AT1G60740       [21]NDQLQTVSVHSIAAGKKVILFGVPGAFTPTCSMSHVPGFIGKAEELKSKGIDEIICFSVNDPFVMKAWGKTY-QENKHV
PRXIIC  AT1G65970       [21]NDQLQTVSVHSIAAGKKVILFGVPGAFTPTCSMSHVPGFIGKAEELKSKGIDEIICFSVNDPFVMKAWGKTY-PENKHV
PRXIIB  AT1G65980       [21]NDQLQTASVHSLAAGKKVILFGVPGAFTPTCSMKHVPGFIEKAEELKSKGVDEIICFSVNDPFVMKAWGKTY-PENKHV
                                                *..***:*::*::                      * :         *
```
