# Supplementary figures and images for: Genome-wide identification, evolution, expression, and alternative splicing profiles of peroxiredoxin genes in cotton
Source: PeerJ. 2021 Jan 18;9:e10685. doi: 10.7717/peerj.10685 (PMC7819121; doi:10.7717/peerj.10685)

Motif ID

LOGO

E-value

Motif 1

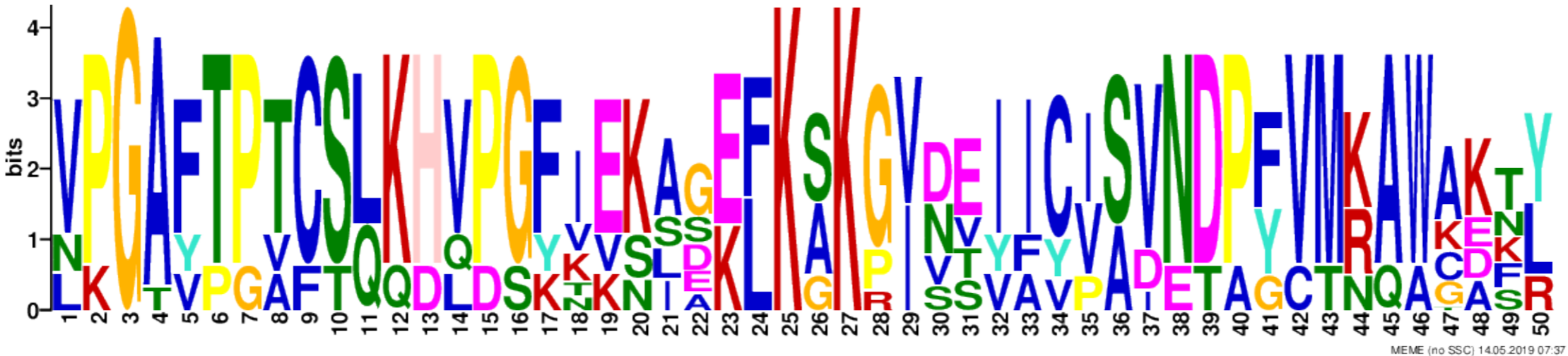

3.6e-1047

Motif 2

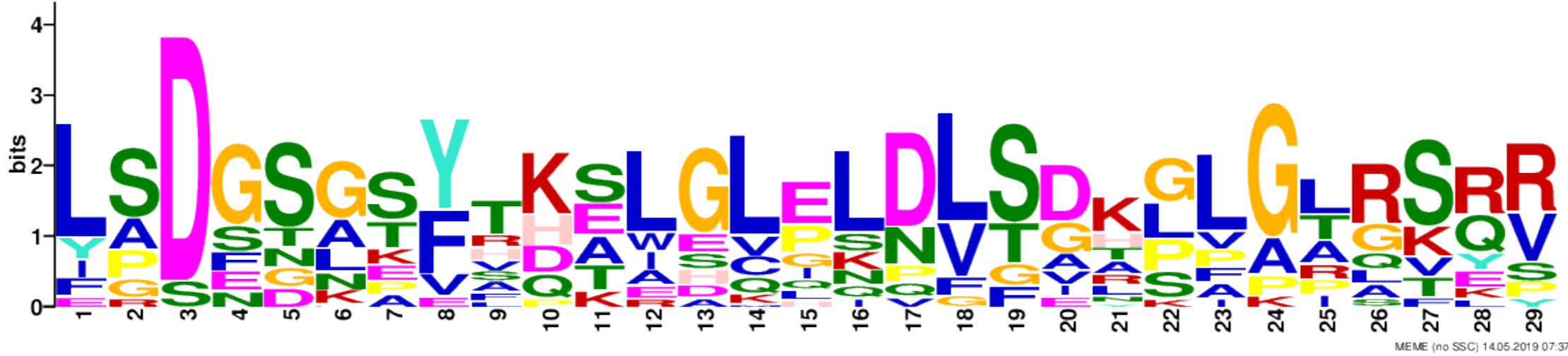

1.8e-432

Motif 3

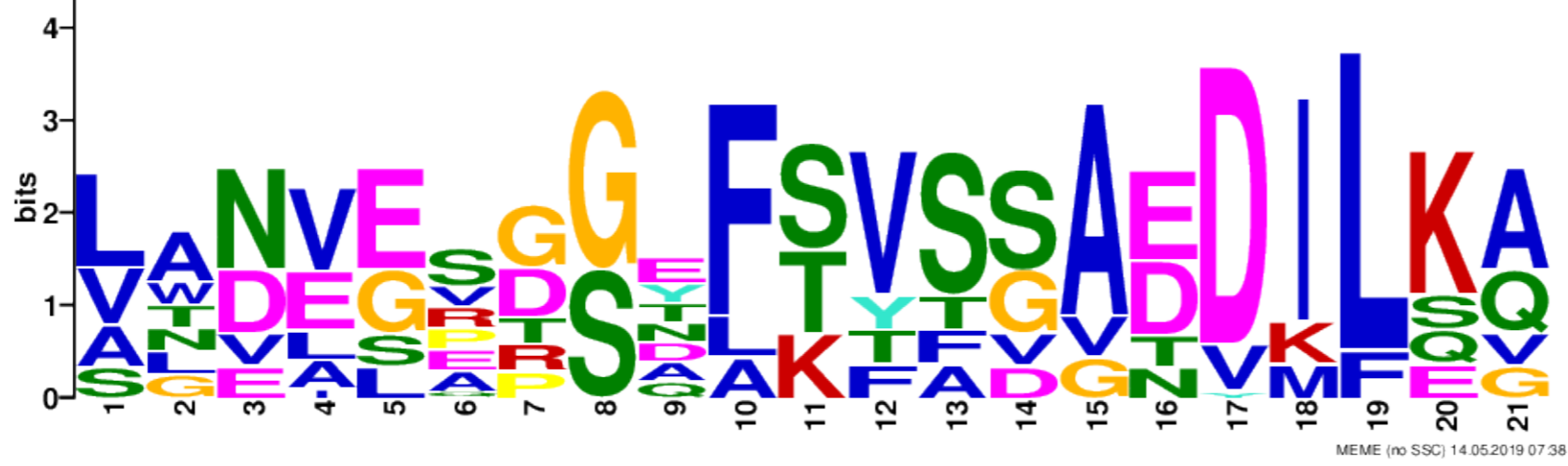

3.2e-297

Motif 4

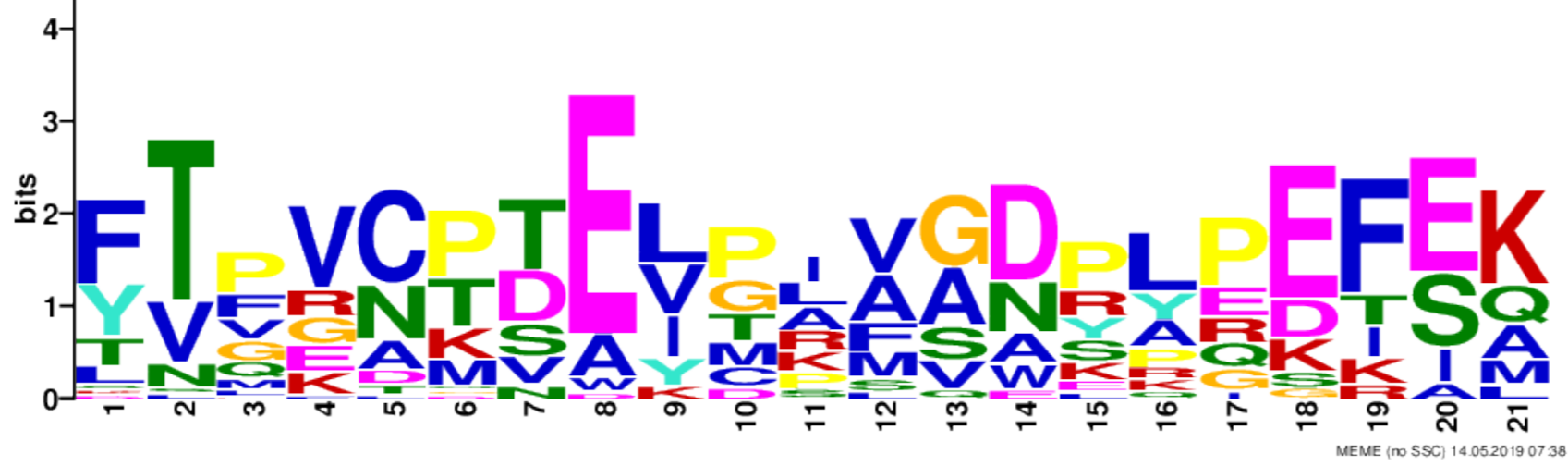

1.2e-242

Motif 5

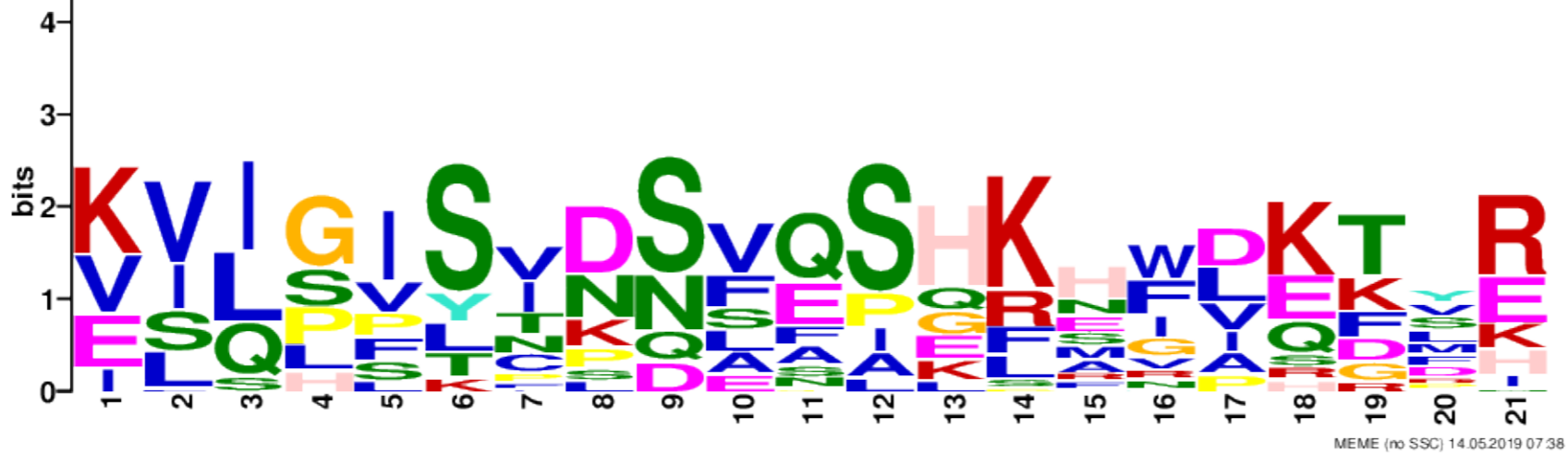

1.2e-212

Motif 6

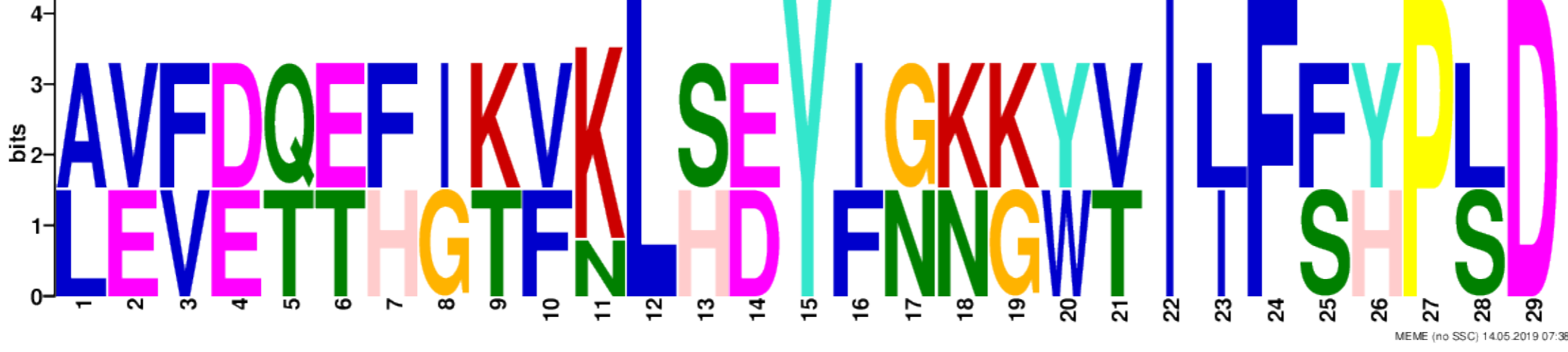

2.5e-195

Motif 7

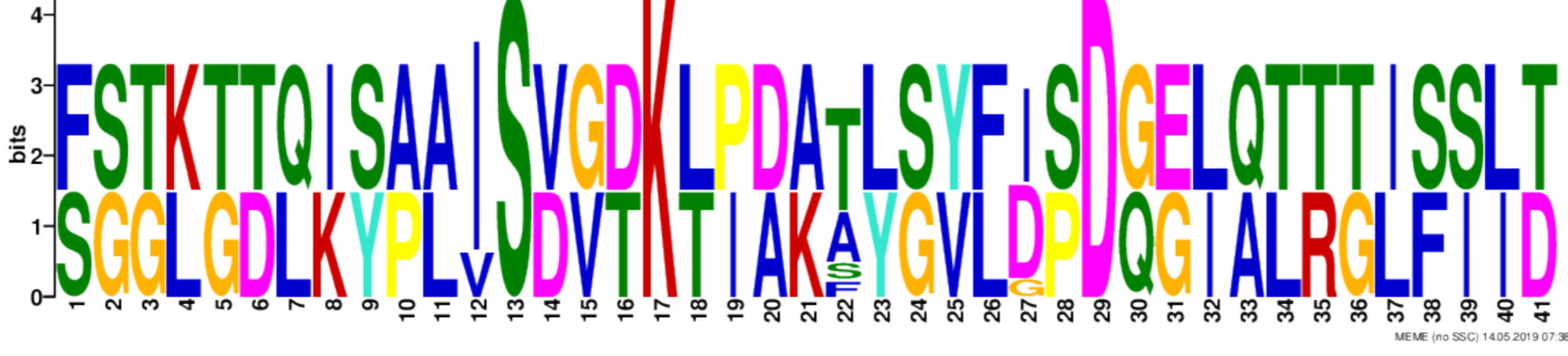

3.7e-155

Motif 8

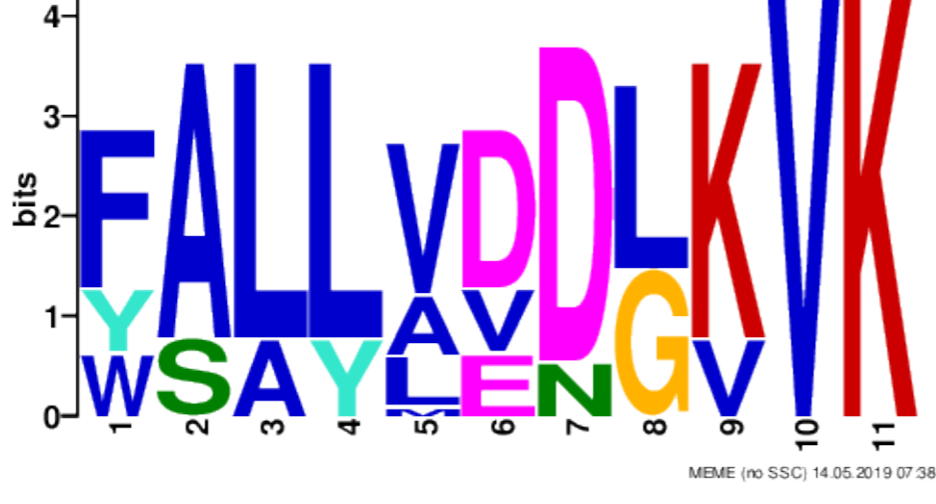

1.2e-110

Motif 9

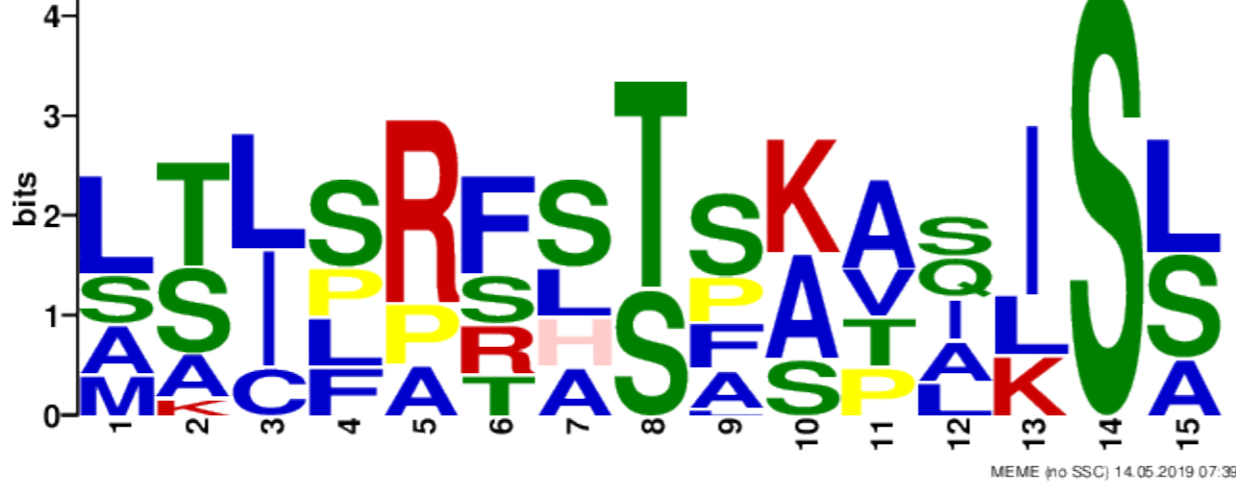

9.9e-092

Motif 10

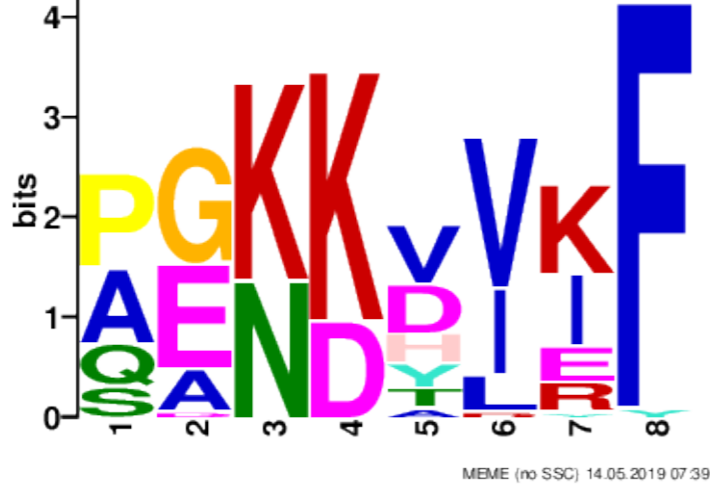

1.5e-071

Supplement: Figure S2 [file peerj-09-10685-s002.pdf]

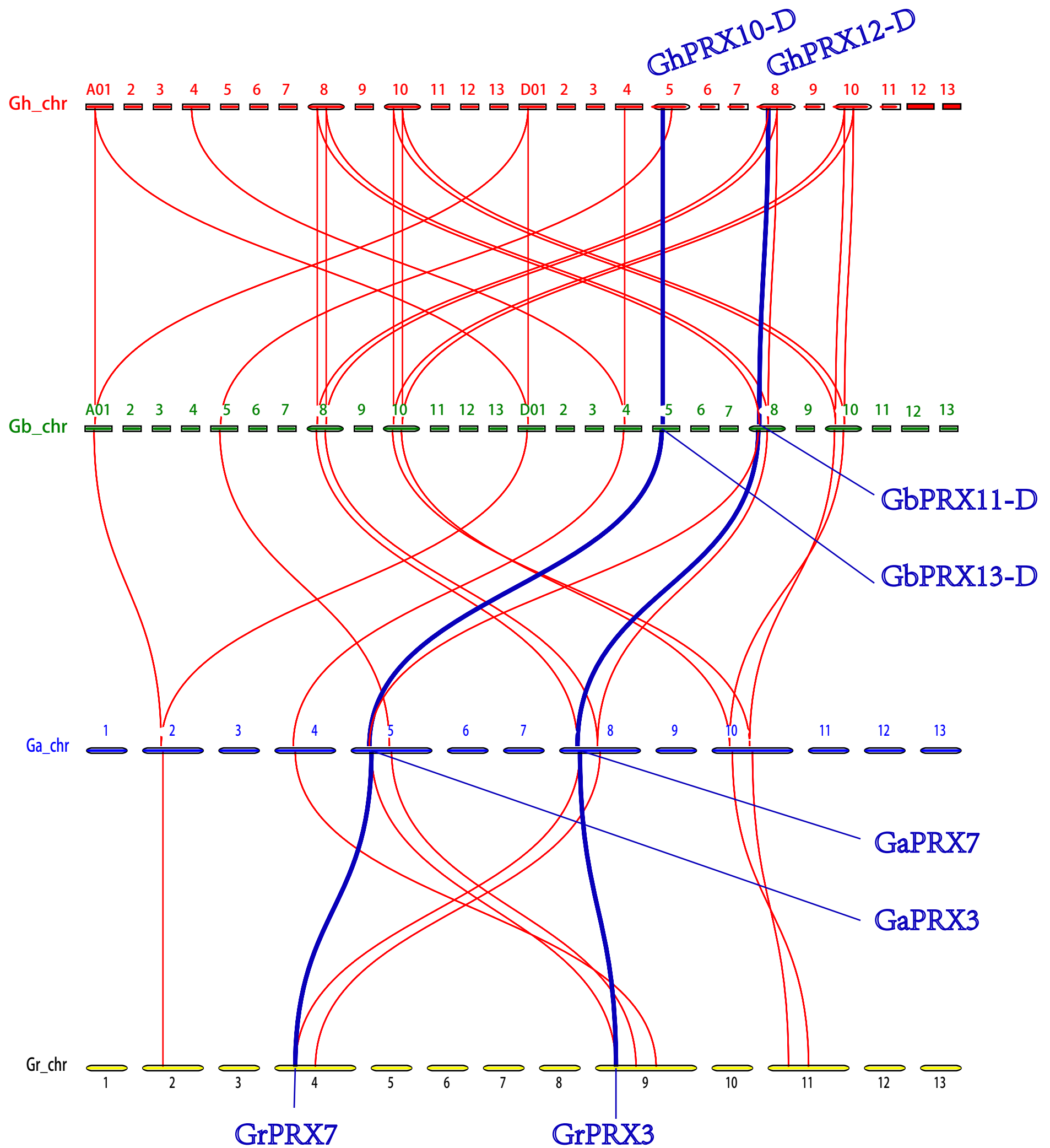

Supplement: Figure S4 — The homology of all Prxs in the four cotton genomes were linked by a red curve, and the Prxs collinearity of segmental duplication were marked by blue. [file peerj-09-10685-s004.pdf]

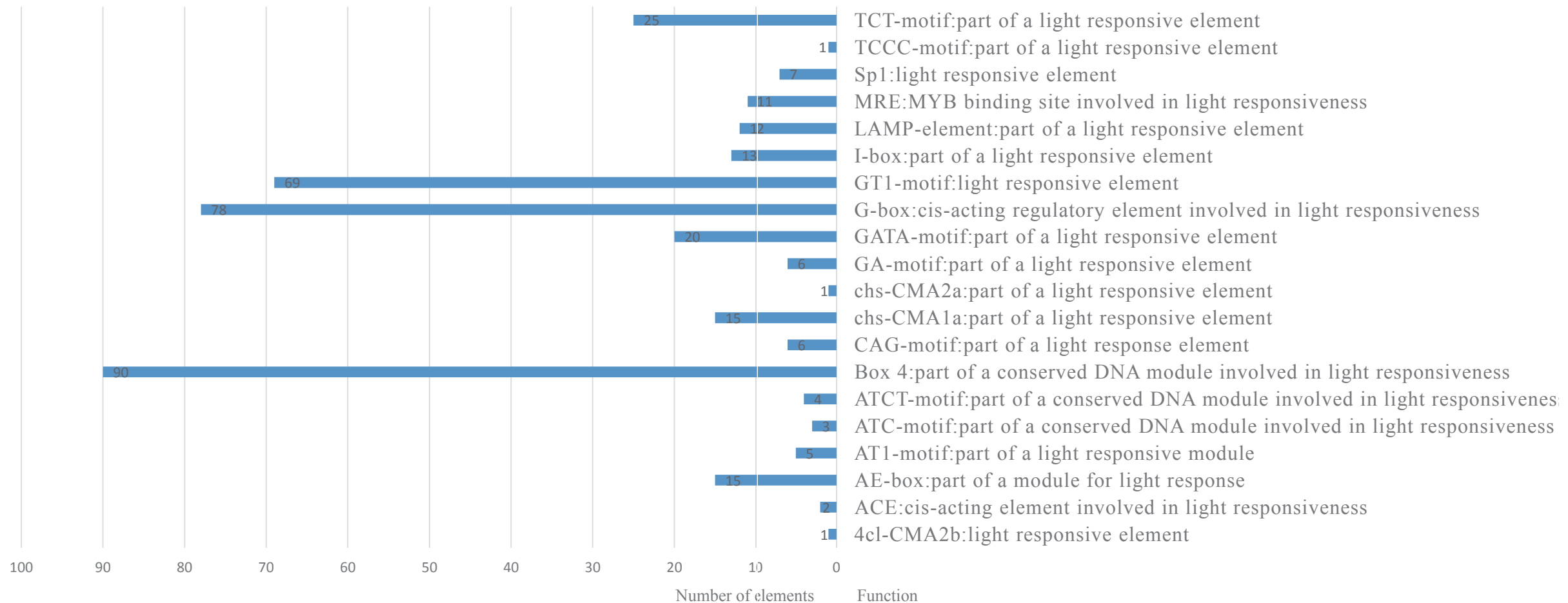

Supplement: Figure S5 [file peerj-09-10685-s005.pdf]
