# Supplementary material for: Genome-wide identification, evolution, expression, and alternative splicing profiles of peroxiredoxin genes in cotton
Source: PeerJ. 2021 Jan 18;9:e10685. doi: 10.7717/peerj.10685 (PMC7819121; doi:10.7717/peerj.10685)
Supplement: Figure S3 [file peerj-09-10685-s003.pdf]

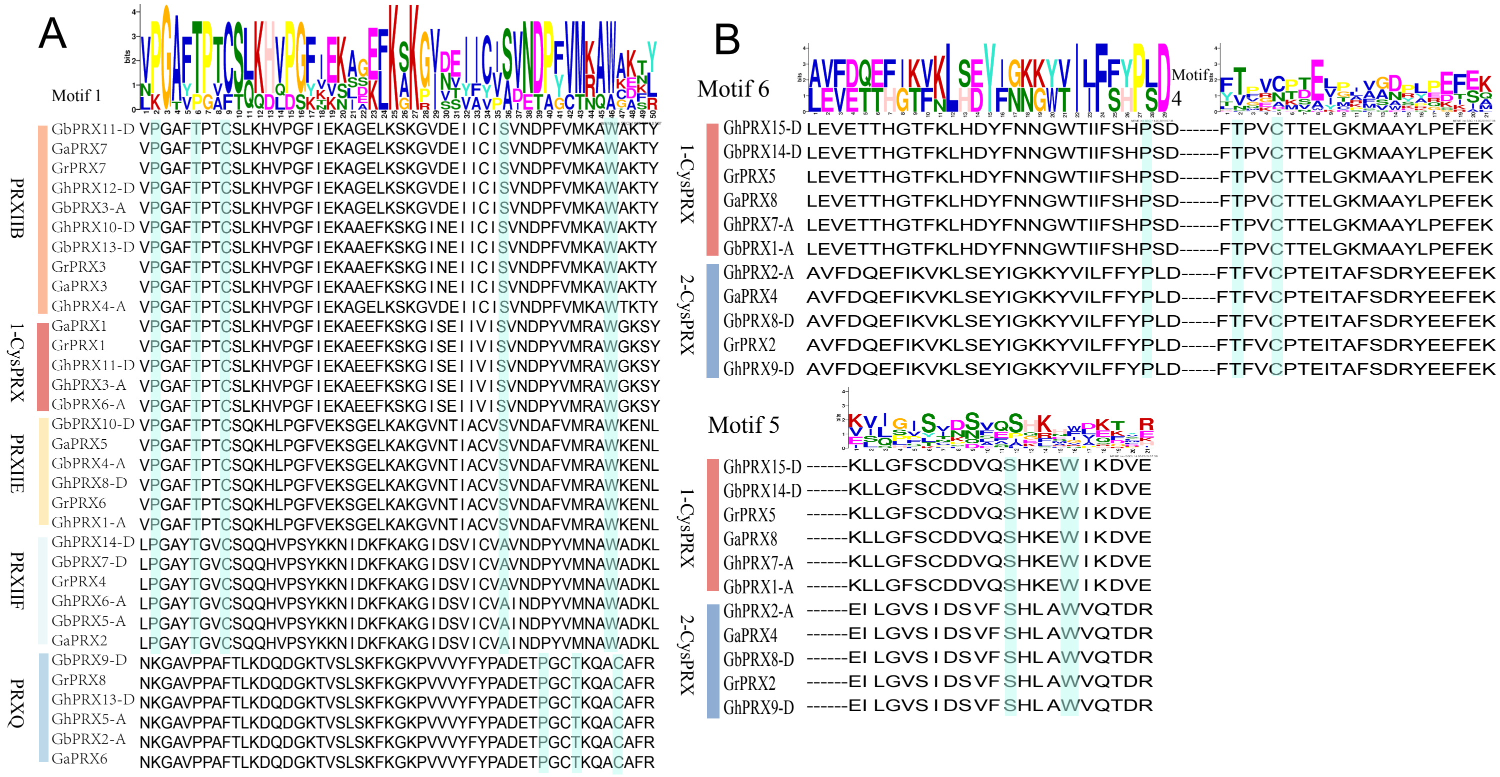
A
Motif 1
PRXIIB
GbPRX11-D VPGAFTPTCSLKHVPGFIEKAGELKSKGVDEIICISVNDPFVMKAWAKTY
GaPRX7 VPGAFTPTCSLKHVPGFIEKAGELKSKGVDEIICISVNDPFVMKAWAKTY
GrPRX7 VPGAFTPTCSLKHVPGFIEKAGELKSKGVDEIICISVNDPFVMKAWAKTY
GhPRX12-D VPGAFTPTCSLKHVPGFIEKAGELKSKGVDEIICISVNDPFVMKAWAKTY
GbPRX3-A VPGAFTPTCSLKHVPGFIEKAGELKSKGVDEIICISVNDPFVMKAWAKTY
GhPRX10-D VPGAFTPTCSLKHVPGFIEKAAEFKSKGINEIICISVNDPFVMKAWAKTY
GbPRX13-D VPGAFTPTCSLKHVPGFIEKAAEFKSKGINEIICISVNDPFVMKAWAKTY
GrPRX3 VPGAFTPTCSLKHVPGFIEKAAEFKSKGINEIICISVNDPFVMKAWAKTY
GaPRX3 VPGAFTPTCSLKHVPGFIEKAAEFKSKGINEIICISVNDPFVMKAWAKTY
GhPRX4-A VPGAFTPTCSLKHVPGFIEKAGELKSKGVDEIICISVNDPFVMKAWTKTY
1-CysPRX
GaPRX1 VPGAFTPTCSLKHVPGFIEKAEEFKSKGISEIIVISVNDPYVMRAWGKSY
GrPRX1 VPGAFTPTCSLKHVPGFIEKAEEFKSKGISEIIVISVNDPYVMRAWGKSY
GhPRX11-D VPGAFTPTCSLKHVPGFIEKAEEFKSKGISEIIVISVNDPYVMRAWGKSY
GhPRX3-A VPGAFTPTCSLKHVPGFIEKAEEFKSKGISEIIVISVNDPYVMRAWGKSY
GbPRX6-A VPGAFTPTCSLKHVPGFIEKAEEFKSKGISEIIVISVNDPYVMRAWGKSY
PRXIIE
GbPRX10-D VPGAFTPTCSQKHLPGFVEKSGELKAKGVNTIACVSVNDAFVMRAWKENL
GaPRX5 VPGAFTPTCSQKHLPGFVEKSGELKAKGVNTIACVSVNDAFVMRAWKENL
GbPRX4-A VPGAFTPTCSQKHLPGFVEKSGELKAKGVNTIACVSVNDAFVMRAWKENL
GhPRX8-D VPGAFTPTCSQKHLPGFVEKSGELKAKGVNTIACVSVNDAFVMRAWKENL
GrPRX6 VPGAFTPTCSQKHLPGFVEKSGELKAKGVNTIACVSVNDAFVMRAWKENL
GhPRX1-A VPGAFTPTCSQKHLPGFVEKSGELKAKGVNTIACVSVNDAFVMRAWKENL
PRXIIF
GhPRX14-D LPGAYTGVCSQQHVPSYKKNIDKFKAKGIDSVICVAVNDPYVMNAWADKL
GbPRX7-D LPGAYTGVCSQQHVPSYKKNIDKFKAKGIDSVICVAVNDPYVMNAWADKL
GrPRX4 LPGAYTGVCSQQHVPSYKKNIDKFKAKGIDSVICVAVNDPYVMNAWADKL
GhPRX6-A LPGAYTGVCSQQHVPSYKKNIDKFKAKGIDSVICVAINDPYVMNAWADKL
GbPRX5-A LPGAYTGVCSQQHVPSYKKNIDKFKAKGIDSVICVAINDPYVMNAWADKL
GaPRX2 LPGAYTGVCSQQHVPSYKKNIDKFKAKGIDSVICVAINDPYVMNAWADKL
PRXQ
GbPRX9-D NKGAVPPAFTLKDQDGKTVSLSKFKGKPVVVYFYPADETPGCTKQACAFR
GrPRX8 NKGAVPPAFTLKDQDGKTVSLSKFKGKPVVVYFYPADETPGCTKQACAFR
GhPRX13-D NKGAVPPAFTLKDQDGKTVSLSKFKGKPVVVYFYPADETPGCTKQACAFR
GhPRX5-A NKGAVPPAFTLKDQDGKTVSLSKFKGKPVVVYFYPADETPGCTKQACAFR
GbPRX2-A NKGAVPPAFTLKDQDGKTVSLSKFKGKPVVVYFYPADETPGCTKQACAFR
GaPRX6 NKGAVPPAFTLKDQDGKTVSLSKFKGKPVVVYFYPADETPGCTKQACAFR
B
Motif 6
Motif 4
1-CysPRX
GbPRX15-D LEVETTHGTFKLHDYFNNGWTIIFSHPSD------FTPVCTTELGKMAAYLPEFEK
GbPRX14-D LEVETTHGTFKLHDYFNNGWTIIFSHPSD------FTPVCTTELGKMAAYLPEFEK
GrPRX5 LEVETTHGTFKLHDYFNNGWTIIFSHPSD------FTPVCTTELGKMAAYLPEFEK
GaPRX8 LEVETTHGTFKLHDYFNNGWTIIFSHPSD------FTPVCTTELGKMAAYLPEFEK
GhPRX7-A LEVETTHGTFKLHDYFNNGWTIIFSHPSD------FTPVCTTELGKMAAYLPEFEK
GbPRX1-A LEVETTHGTFKLHDYFNNGWTIIFSHPSD------FTPVCTTELGKMAAYLPEFEK
2-CysPRX
GhPRX2-A AVFDQEFIKVKLSEYIGKKYVILFFYPLD-----FTFVCPTEITAFSDRYEEFEK
GaPRX4 AVFDQEFIKVKLSEYIGKKYVILFFYPLD-----FTFVCPTEITAFSDRYEEFEK
GbPRX8-D AVFDQEFIKVKLSEYIGKKYVILFFYPLD-----FTFVCPTEITAFSDRYEEFEK
GrPRX2 AVFDQEFIKVKLSEYIGKKYVILFFYPLD-----FTFVCPTEITAFSDRYEEFEK
GhPRX9-D AVFDQEFIKVKLSEYIGKKYVILFFYPLD-----FTFVCPTEITAFSDRYEEFEK
Motif 5
1-CysPRX
GhPRX15-D ------KLLGFSCDDVQSHKEWIKDVE
GbPRX14-D ------KLLGFSCDDVQSHKEWIKDVE
GrPRX5 ------KLLGFSCDDVQSHKEWIKDVE
GaPRX8 ------KLLGFSCDDVQSHKEWIKDVE
GhPRX7-A ------KLLGFSCDDVQSHKEWIKDVE
GbPRX1-A ------KLLGFSCDDVQSHKEWIKDVE
2-CysPRX
GhPRX2-A ------EILGVSIDSVFSHLAWVQTDR
GaPRX4 ------EILGVSIDSVFSHLAWVQTDR
GbPRX8-D ------EILGVSIDSVFSHLAWVQTDR
GrPRX2 ------EILGVSIDSVFSHLAWVQTDR
GhPRX9-D ------EILGVSIDSVFSHLAWVQTDR
