# Supplementary material for: Genome-wide identification, evolution, expression, and alternative splicing profiles of peroxiredoxin genes in cotton
Source: PeerJ. 2021 Jan 18;9:e10685. doi: 10.7717/peerj.10685 (PMC7819121; doi:10.7717/peerj.10685)
Supplement: File S2 [file peerj-09-10685-s012.zip › Supplemental_File_2/Readme.doc]

Sample-0h.txt is real-time quantitative PCR file without treatment 0h sample. Sample-N-1h.txt, Sample-N-3h.txt, Sample-N-6h.txt and Sample-N-12h.txt are real-time quantitative PCR files of 1 h, 3 h, 6 h and 12 h samples treated by NaCl. Sample-S-1h.txt, Sample-S-3h.txt, Sample-S-6h.txt and Sample-S- 12h.txt are real-time quantitative PCR documents of 1 h, 3 h, 6 h and 12 h samples treated by SA. Sample-P-1h.txt, Sample-P-3h.txt, Sample-P-6h.txt and Sample-P-12h.txt are real-time quantitative PCR documents of 1 h, 3 h, 6 h and 12 h samples treated by PEG-6000. Control-leaves-1,3.txt is 1h and 3h untreated control leaves sample files, Control-leaves-6,12.txt is 6h and 12h untreated control leaves sample files. Control-roots-1,3.txt is 1h and 3h untreated control roots sample files, Control-roots-6,12.txt is 6h and 12h untreated control roots sample files.

A is GhPRX1-A. B, it's GhPRX2-A. C, it's GhPRX4-A. D, it's GhPRX6-A. E, it's GhPRX7-A. F, it's GhPRX8-D. G, it's GhPRX9-D. H, it's GhPRX14-D. J, it's GhPRX16-D. K, it's GhPRX3-A. L is GhPRX10-D. M is GhPRX16-D. N is GhPRX5-A. O is GhPRX11-D. P is GhPRX13-D. 1 -9 is 3 leaves samples with 9 mechanical repeats, 10-18 is 3 roots samples with 9 mechanical repeats, A19-A21, B19-B21 and C19-C21 are the UBQ7 gene internal reference of 3 leaves samples, and D19-D21, E19 -E21 and F19-F21 are UBQ7 gene internal reference of 3 root samples.
